# Molecular identification of *Trichuris trichiura* and *Hymenolepis diminuta* in long-tailed macaques (*Macaca fascicularis*) in Lopburi, Thailand

**DOI:** 10.14202/vetworld.2021.884-888

**Published:** 2021-04-13

**Authors:** Wanat Sricharern, Tawin Inpankaew, Sarawan Kaewmongkol, Thitichai Jarudecha, Natnaree Inthong

**Affiliations:** 1 Center for Agricultural Biotechnology, Kasetsart University, Kamphaeng Saen Campus, Nakhon Pathom, Thailand; 2 Center of Excellence on Agricultural Biotechnology, Science and Technology Postgraduate Education and Research Development Office, Commission on Higher Education, Ministry of Education, Science, Research Innovation (AG-BIO/PERDO-CHE), Bangkok Thailand; 3 Department of Veterinary Technology, Faculty of Veterinary Technology, Kasetsart University, Bangkok, Thailand; 4 Department of Parasitology, Faculty of Veterinary Medicine, Kasetsart University, Bangkok, Thailand

**Keywords:** *Hymenolepis diminuta*, long-tailed macaque, *Macaca fascicularis*, Thailand, *Trichuris trichiura*

## Abstract

**Background and Aim:**

*Trichuris trichiura* and *Hymenolepis diminuta* are helminthic intestinal parasites that infect humans and other animals, including non-human primates. However, molecular detection of these parasites remains scarce in long-tailed macaques (*Macaca fascicularis*), which coexist with human communities in Thailand. Thus, this study aimed to molecularly confirm the occurrence of *Trichuris* spp. and *Hymenolepis* spp. infection and determine the species of both parasites that were found in long-tailed macaques.

**Materials and Methods:**

A total of 200 fecal samples were randomly collected from long-tailed macaques living in Lopburi, Thailand, and tested based on polymerase chain reaction (PCR) assays for *Trichuris* spp. and *Hymenolepis* spp. infections. The PCR products were submitted for DNA purification and sequencing. Phylogenetic analysis was performed using the maximum likelihood method.

**Results:**

Of 200 tested samples, three (1.5%) were positive for *Trichuris* spp. Sequence analysis of all positive samples revealed the presence of *T. trichiura*, while eight samples (8/200, 4%) positive for *Hymenolepis* spp. were classified as *H. diminuta*. No significant associations were found between parasite infection and sex of macaques.

**Conclusion:**

This study revealed that long-tailed macaques harbor *T. trichiura* and *H. diminuta*. These results suggested that local residents and tourists must pay attention to limiting contact with long-tailed macaques and take hygienic precautions to reduce the risk of zoonotic and anthroponotic transmission of these parasites between humans and long-tailed macaques.

## Introduction

Parasitic infections caused by intestinal helminths in humans are ubiquitous in distribution and constitute a major part of neglected tropical diseases [1]. *Trichuris* spp. worms or whipworms are soil-transmitted nematode parasites that can be an important cause of inflammation of the cecum and large intestine, bloody diarrhea, and chronic iron deficiency anemia in humans [2,3]. The infection develops by ingestion of embryonated eggs of the parasite, which contaminate the soil or food [2]. There are approximately 100 species in the genus *Trichuris* that infect a number of hosts, such as humans (*Trichuris trichiura*), pigs (*Trichuris suis*), dogs (*Trichuris vulpis*), and rodents (*Trichuris muris*). However, three species (*T. trichiura*, *T. suis*, and *T. vulpis*) are considered zoonotic parasites. Although *T. suis* and *T. vulpis* infections have been reported in humans, these parasites generally cause attenuated infections and rarely develop sexual maturity in humans [2,4]. Moreover, a study reported that non-human primates (NHPs) share whipworms with humans [3]. *Trichuris* spp. have been detected in several species of NHP in many countries, including Malaysia, Japan, China, Italy, Uganda, Gabon, and Thailand [5-12].

*Hymenolepis diminuta* (rat tapeworm) is a tapeworm of rodents that are present worldwide [13]. Human infections with this parasite have been reported in 80 countries from 1810 to 2018, including seven cases in Thailand [14]. Humans are infected by ingesting cysticercoid larvae that are harbored by beetles, fleas, caterpillars, and other insects [13]. Most reported cases are asymptomatic. Common symptoms are primarily associated with intestinal symptoms, such as abdominal pain and diarrhea. However, remittent fever, diffuse cutaneous itching, and arthromyalgia have been reported [15,16]. *Hymenolepis* spp. have been detected in NHP from Peru and white-headed capuchin monkeys from Ecuador [17,18].

The long-tailed macaque (*Macaca fascicularis*), also known as the crab-eating macaque or cynomolgus macaque, is the most commonly observed species of NHPs that inhabit many regions of Thailand [19]. Lopburi province is one of the most famous tourist attractions in Central Thailand, where these macaques live and share the environment with local residents and tourists. Macaques can carry numerous pathogens that are potentially zoonotic to humans, more specifically gastrointestinal parasites that cause the most common diseases found in NHPs [20]. Transmission of parasites from animals to humans can occur due to humans and NHPs sharing the same ecosystem [17].

The investigation of *Trichuris* spp. and *Hymenolepis* spp. infection in long-tailed macaques is necessary to determine their potential as a public health threat. However, there are still extremely few studies on both these parasites in long-tailed macaques, especially *Hymenolepis* spp. infection in Thailand. Furthermore, the conventional diagnosis of gastrointestinal helminth infections in NHP is based on the detection of parasitic eggs in feces, and consideration of only egg morphology may be insufficiently reliable for species identification [6].

Therefore, the current study aimed to report the occurrence of *Trichuris* spp. and *Hymenolepis* spp. infection and determine the species of both parasites found in long-tailed macaques using molecular methods.

## Materials and Methods

### Ethical approval

This research was approved by the Animal Ethics Committee of Kasetsart University, Bangkok, Thailand (ACKU59-VTN-011).

### Study period and location

The blood samples were collected in April 2014 from long-tailed macaques in Lopburi province, Thailand. Laboratory analysis was carried out in the Faculty of Veterinary Technology, Kasetsart University, Thailand.

### Samples collection and study area

In April 2014, convenience sampling was used to collect fecal samples from 200 free-ranging long-tailed macaques (86 female and 114 male macaques) at the San Phra Kan Shrine, Lopburi province, Central Thailand. Sex was identified by observation of the external genital organ of each macaque. The feces were immediately collected from the ground after defecation of macaques and kept cool during transportation. All fecal specimens were washed with distilled water and sieved to eliminate the large sediment from the samples before storage of fecal suspensions at −20°C to perform freeze/thaw cycle of the samples before subjecting to DNA extraction [13].

### Molecular analysis and DNA sequencing

DNA was extracted from 200 mL of fecal suspension using the E.Z.N.A.^®^ stool DNA extraction kit (OMEGA Bio-tek Inc., USA), according to the manufacturer’s instructions. Extracted DNA samples were stored at −40°C until use in molecular analysis. The DNA samples were assayed for *Trichuris* spp. and *Hymenolepis* spp. For the confirmation of *Trichuris* spp., the genomic DNA was amplified, targeting the internal transcribed spacer region 1 (ITS-1) as per Ghai *et al*. [10]. The primary polymerase chain reaction (PCR) used the primers ExternalITS1_*Trichuris*-1417F (5’-AGGGACCAGCGACACTTTC-3’) and ExternalITS1_*Trichuris*-2505R (5’-GAGTGTC ACGT CGTTCTTCAAC-3’). The thermal cycling profile for primary PCR was as follows: 94°C for 5 min; 40 cycles of 94°C for 60 s, 61°C for 30 s, 72°C for 75 s, and a final extension at 72°C for 10 min. The secondary PCR used the primers InternalITS1_*Trichuris*-1567F (5’-GTTCTCGTGACTGGGAC-3’) and InternalITS1_*Trichuris*-2462R (5’-CTACGAGCCAAGTGATCC-3’). The nested PCR conditions consisted of the following: 94°C for 60 s, followed by 35 cycles of 94°C for 30 s, 55°C for 30 s, 72°C for 75 s, and a final extension at 72°C for 10 min. The expected sizes of target amplicons were approximately 1088 and 895 bp for the primary and nested PCR, respectively.

For *Hymenolepis* spp., the amplicon of the partial 18S rRNA gene was amplified using the procedure of Olson *et al*. [21] with some modifications. The PCR used the primers Hn107F (5’-GGGAATGGGTGCACTTATTAGA-3’) and Hn312R (5’-GTTATCACCATGGTAGGCAGGT-3’), resulting in approximately 160 bp amplicons. The PCR cycling conditions were as follows: 94°C for 5 min; 35 cycles of 94°C for 30 s, 56°C for 30 s, and 72°C for 75 s; and a final extension at 72°C for 10 min.

The PCR products were analyzed using 1.2% (w/v) agarose gel electrophoresis with TAE buffer and visualized under ultraviolet transillumination after staining the nucleic acid with GelStar^®^ (Cambrex BioScience, USA). The positive samples of the correct size for the ITS-1 of *Trichuris* spp. and the partial 18S rDNA fragment of *Hymenolepis* spp. were submitted for DNA purification and sequencing.

### Phylogenetic analysis

The ITS-1 sequences obtained from *Trichuris* spp. in this study were aligned with sequences available in GenBank, namely, AJ781762, GQ352553, GQ352555, GQ352558, KC877992, MH390365, and MN447320, and the 18S rRNA gene sequences obtained from *Hymenolepis* spp. were compared with published sequences, including AF124475, AF286983, AY193875, JX310720, and KX454312 using the BLAST program of the National Center for Biotechnology Information.

Phylogenetic analysis of the nucleotide sequences was performed using the maximum likelihood method based on the Kimura two-parameter model in the MEGA 7 software (The Biodesign Institute, Tempe, AZ, USA) (http://www.megasoftware.net). The reliability of the reconstructed phylogenies was assessed using the bootstrap method with 1000 replicates.

### Statistical analysis

Statistical analysis was performed using Fisher’s exact test when the data did not comply with the central limit theorem and proportion test (Z test) when the data complied with the central limit theorem to determine the association between *Trichuris* spp. and *Hymenolepis* spp. infections and host sex. p < 0.05 indicated statistical significance.

## Results

Of 200 fecal samples, 3 (1.5%) were positive for *Trichuris* spp. based on the nested PCR analysis of the ITS-1 of the ribosomal DNA complex. The amplicon size retrieved in positive samples was approximately 895 bp. The positive samples consisted of one male and two female macaques (Table-1). No significant association was observed between *Trichuris* spp. Infection and sex of macaques based on Fisher’s exact test (p=0.578).

**Table-1:** *Trichuris trichiura* and *Hymenolepis diminuta* infection in long-tailed macaques (*Macaca fascicularis*).

Gender	Number of animals	Positive samples (%)	

		*Trichuris trichiura*	*Hymenolepis diminuta*
Male	114	1 (0.87)	5 (4.39)
Female	86	2 (2.33)	3 (3.49)
Total	200	3 (1.50)	8 (4.00)

**Figure-1:**
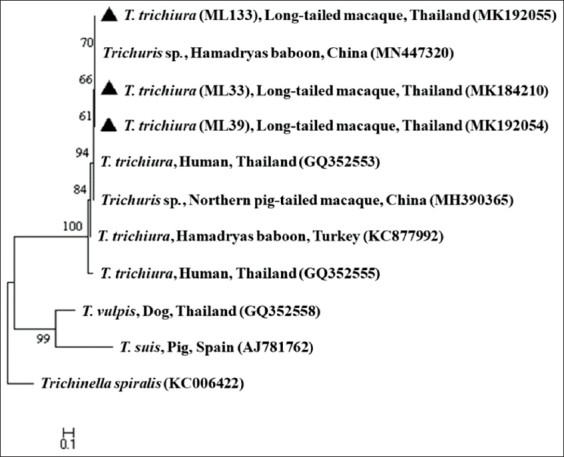
Phylogenetic tree based on internal transcribed spacer region 1 sequences of *Trichuris trichiura* based on the maximum likelihood method. Bootstrap percentages are shown at nodes. *Trichinella spiralis* is the outgroup. Isolates obtained in the current study are coded using triangular symbols.

Sequence analysis of all positive samples revealed the presence of *T. trichiura*. All of these sequences shared 98-99% identity with the sequence of *T. trichiura* isolate TH1 obtained from a human in Thailand (accession number, GQ352553). The nucleotide sequences of the ITS-1 of *T. trichiura* were deposited in the GenBank™ databases under the following accession numbers: MK184210, MK192054, and MK192055. Moreover, the phylogenetic tree based on the ITS-1 of these positive samples revealed that the sequences of *Trichuris* spp. in this study were positioned in the same cluster as *T. trichiura* in humans (GQ352553) and *Trichuris* spp. in other NHPs (MN447320 and MH390365) (Figure-1).

Based on the PCR analysis of the partial 18S rRNA gene, amplicons (160 bp) consistent with *Hymenolepis* spp. were detected in 4% (8/200) of tested fecal samples. The infection rate in male macaques was 4.39% (5/114), which was higher than the infection rate in female macaques (3.49%, 3/86) (Table-1). However, no significant association was found between *Hymenolepis* spp. infection and sex of the host using the proportion test (p=0.748).

**Figure-2:**
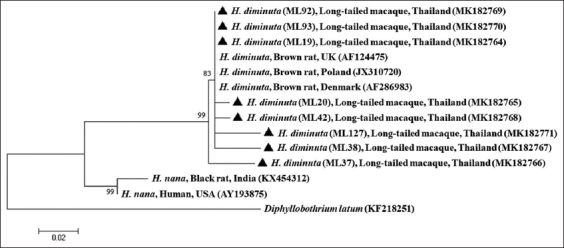
Phylogenetic tree based on partial 18S rRNA gene sequences of *Hymenolepis diminuta* based on the maximum likelihood method. Bootstrap percentages are shown at nodes. *Diphyllobothrium latum* is the outgroup. The isolates obtained in the current study are coded using triangular symbols.

The sequence analysis of the positive samples of *Hymenolepis* spp. revealed that all of these samples were characterized as *H. diminuta*. All sequences had 97-100% similarity to *H. diminuta* isolated from *Rattus norvegicus* (brown rat) in Poland and Denmark (GenBank accession numbers, JX310720 and AF286983). The nucleotide sequences of the partial 18S rDNA of *H. diminuta* in the current study were also submitted to the GenBank™ databases under the following accession numbers: MK182764–MK182771. The 18S rRNA gene nucleotide sequences of *H. diminuta* detected in long-tailed macaques were most closely related to *H. diminuta* in *R. norvegicus* in the tree (Figure-2).

## Discussion

The prevalence of *Trichuris* spp. in this investigation was identified at a low rate (1.5%) based on nested PCR assay of the fecal sample of long-tailed macaques in Thailand. Compared with the current findings, higher infection rates in different species of NHP have been reported in several studies from Africa, including 6.1% (9/147) in wild galagos in Gabon [11], 16.4% (23/140) in baboons in Uganda [9], and 13.3% (4/30), 14.3% (6/42), and 88.9% (24/27) in chimpanzees, gray-cheeked mangabeys, and olive baboons, respectively, in Uganda [10]. Moreover, some studies from Asia have also described higher prevalence rates, including 20.5% (686/3349) in 34 NHP species in China [7], 23.1% (71/308) in 12 NHP species in Malaysia [5], and 82.1% in proboscis monkeys in Malaysia [20]. In Thailand, a higher prevalence rate (20/102, 19.6%) of *Trichuris* spp. infection has been previously reported in long-tailed macaques in Maha Sarakham province, Northeast Thailand, using the formalin-ethyl acetate sedimentation technique [12]. The low infection rate of *T. trichiura* in the current study might have been caused by collection of a single fecal sample from each animal, since parasitic eggs of soil-transmitted helminths are only intermittently shed in the feces of the host, particularly in mild infection [22].

*Trichuris* spp. found in the current study were identified as *T. trichiura* based on sequence and phylogenetic analyses, as were observed in wild Japanese macaques (*Macaca fuscata*) from Japan [6] and ring-tailed lemurs from Italy [8].

The results of the current study suggested that proper sanitation and personal hygiene should be encouraged for local residents and tourists to reduce the risk of zoonotic transmission of *T. trichiura* from NHPs, since there has been a report of the circulation of *Trichuris* species between humans and NHPs [3]. Furthermore, this parasite is a major zoonotic intestinal helminth in Southeast Asia, and the highest prevalence of human trichuriasis has been reported in this region [1].

In the current study, *H. diminuta* was identified at a relatively low rate in long-tailed macaques, which was similar to that in a previous study on NHPs with 2.3% (9/382) in *Aotus nancymaae*, 1.8% (4/221) in *Aotus vociferans*, and 1% (1/90) in *Saguinus mystax* in Peru [17]. Conversely, a high prevalence of *Hymenolepis* spp. was reported in white-headed capuchin monkeys (38.46%, 10/26) from Ecuador [18].

The molecular characterization of both parasites is recommended since microscopic examination is insufficient for species identification. Precise diagnosis of zoonotic parasites is necessary for disease surveillance, control, and elimination programs [1]. Although the prevalence of these parasites in the current study was low, the identification of both helminths might indicate the potential of zoonotic transmission in this area.

Further studies should include surveys of *Trichuris* spp. and *Hymenolepis* spp. in various NHPs in other locations in Thailand. Characterization of these parasites from humans and NPHs living in a shared area is needed to quantify the transmission between humans and NHPs. Moreover, investigations of *H. diminuta* in rodents in the same location as long-tailed macaques seem necessary because rodents are the main definitive host of this parasite [1], and studies have found this parasite in rodents in Mexico and Malaysia [14,23].

## Conclusion

The current study identified a low infection rate of *T. trichiura* and *H. diminuta* in long-tailed macaques in Lopburi province, Thailand. However, both parasites are pathogenic to humans. Therefore, the results from this investigation suggested that contact should be reduced between long-tailed macaques and humans. Moreover, appropriate personal hygiene should be maintained by local residents and tourists during the handling of these macaques to reduce the zoonotic and anthroponotic transmission of these parasites.

## Authors’ Contributions

WS designed and conducted the study, interpreted the results, and drafted the manuscript. TI revised and finalized the manuscript for submission. SK and NI supervised the molecular analyses. TJ interpreted the results. All authors read and approved the final manuscript.
